# Species-specific PCR assay for the detection of *Babesia odocoilei*

**DOI:** 10.1177/10406387211032927

**Published:** 2021-09-22

**Authors:** Hilary J. Burgess, Betty P. Lockerbie, Lisanework E. Ayalew, Antonia Dibernardo, Kristýna Hrazdilová, David Modry, Trent K. Bollinger

**Affiliations:** Department of Veterinary Pathology, Western College of Veterinary Medicine, University of Saskatchewan, Saskatoon, Saskatchewan, Canada; Department of Veterinary Pathology, Western College of Veterinary Medicine, University of Saskatchewan, Saskatoon, Saskatchewan, Canada; Department of Veterinary Pathology, Western College of Veterinary Medicine, University of Saskatchewan, Saskatoon, Saskatchewan, Canada; National Microbiology Laboratory, Public Health Agency of Canada, Winnipeg, Manitoba, Canada; CEITEC-VFU, University of Veterinary and Pharmaceutical Sciences Brno, Brno, Czech Republic; CEITEC-VFU, University of Veterinary and Pharmaceutical Sciences Brno, Brno, Czech Republic; Department of Botany and Zoology, Faculty of Science, Masaryk University, Brno, Czech Republic; Biology Centre, Institute of Parasitology, Czech Academy of Sciences, České Budějovice, Czech Republic; Department of Veterinary Pathology, Western College of Veterinary Medicine, University of Saskatchewan, Saskatoon, Saskatchewan, Canada; Canadian Wildlife Health Cooperative, Western College of Veterinary Medicine, University of Saskatchewan, Saskatoon, Saskatchewan, Canada

**Keywords:** *Babesia odocoilei*, cervids, PCR, specificity

## Abstract

We developed a PCR assay for the detection of *Babesia odocoilei* based on the 18S rRNA gene. Multiple specimens of *B. odocoilei* were examined, and the assay consistently produced a small specific PCR product of 306 bp. The PCR assay was also challenged with DNA from 13 other *Babesia* species and 2 *Theileria* species, originating from 10 different host species; however, nonspecific DNA amplification and multiple banding patterns were observed, and the amplicon banding patterns varied between different isolates of the same species. Sensitivity was determined to be 6.4 pg of DNA, and an estimated 0.0001% parasitism. This assay can be utilized for species-specific differential detection of *B. odocoilei*.

*Babesia odocoilei* is an intraerythrocytic protozoan parasite, transmitted by *Ixodes* spp. ticks, that has been identified in cervids within Canada since 2013.^13,16^ *B. odocoilei* infection can manifest as subclinical infection, chronic debilitating disease, or acute fatal hemolytic anemia.^3,6,17^ Diagnosis can be achieved through visualization of organisms in peripheral blood smears, detection of antibodies using serology, and PCR testing. PCR has the advantage of high sensitivity, and the ability to precisely identify the organism, which is particularly important when there is the possibility of infection with morphologically similar organisms such as *Theileria* spp. or other species of *Babesia.*^1,2,19,20^

To date, *B. odocoilei* is the only piroplasm known to infect cervids in Canada, but this is not the case in other countries in which several different *Babesia* species must be considered, and coinfections are common.^9^ Cervid babesiosis is considered an emerging disease in Canada. The geographic distribution in wild and captive cervids, and the role of non-cervid hosts in regional *B. odocoilei* epidemiology, is uncertain.^14^ Surveillance data are limited, leaving many gaps in our understanding of the risk to captive and free-ranging cervids in Canada.^14^ A specific test for *B. odocoilei* that could be used on a variety of samples, including formalin-fixed tissue, would facilitate both prospective and retrospective surveillance of this emerging pathogen in Canada.

We evaluated sensitivity and confirmed the specificity of a 306-bp PCR assay for *B. odocoilei* when challenged with DNA from a variety of *Babesia* and *Theileria* spp. For cervids, fresh or frozen blood, spleen, or velvet antler are generally preferred for PCR testing, with follow-up sequencing confirming the specificity of the test.^10^ However, there are many circumstances in which fresh or frozen tissue may not be readily available and testing is limited to formalin-fixed samples. It has been well established that formalin causes chemical modifications and crosslinking of proteins, thereby reducing amplicon length or rendering samples unsuitable for PCR.^12^ With this in mind, a set of primers has been designed to only anneal with the *B. odocoilei* sequence, providing specificity, as well as producing a smaller product (306 bp) more suitable to the reduced amplicon length often associated with formalin fixation.^10^ Achieving specificity of this assay was important because the ability to confirm *Babesia* species through sequencing was lost given the smaller product size and loss of the annealing site during trimming and editing. In a previous study, this PCR was shown to differentiate known *B. odocoilei*–positive and –negative samples, and was found to be effective in detecting *B. odocoilei* in tissues stored frozen at −20°C for 1 y, and in 10% neutral-buffered formalin for up to 6 mo.^10^

DNA samples (extracted from mammalian blood [*n* = 35] and ticks [*n* = 10]) and reference strains (*n* = 3) of a variety of *Babesia* and *Theileria* spp. were procured from the Department of Parasitology, Institute of Genetics and Microbiology, University of Wrocław (Wrocław, Poland); the Department of Veterinary Pathobiology, Texas Veterinary Medical Center, Texas A&M University (College Station, TX, USA); and the authors’ laboratories to assess the specificity of the PCR assay (Table 1).

**Table 1.** Host, geographic origin, method of identification, GenBank accession, and result of our PCR assay of 48 samples of *Babesia* and *Babesia*-like organisms isolated and purified from mammalian blood (*n* = 35), tick (*n* = 10) samples, or obtained as reference strains (*n* = 3).

Organism species	Host animal/origin	Geographic origin	Method of identification/GenBank accession	306-bp PCR result
*B. bigemina*	Bovid	Mexico	PCR^8^	−
	Bovid	St. Croix, U.S. Virgin Islands	PCR^8^; MH050356	−
*B. bovis*	Bovid	Mexico	PCR^8^	−
*B. caballi*		USDA reference strain		−
*B. canis*	Red fox	Ruszów Forestry, Poland	PCR^9^	−
	Red fox	lower Silesian District, Poland	PCR^9^	−
	Dog	South Moravia, Czech Republic	PCR^9^	−
	Dog	Central Bohemia, Czech Republic	PCR^9^	−
*B. capreoli*	Red deer	Hradiště, Czech Republic	PCR^9^; MG344751	−
	Red deer	Hradiště, Czech Republic	PCR^9^; MG344782	−
	Roe deer	Hradiště, Czech Republic	PCR^9^; MG344746	−
	Roe deer	Libavá, Czech Republic	PCR^9^; MG344754	−
	Roe deer	Libavá, Czech Republic	PCR^9^; MG344797	−
	Sika deer	Hradiště, Czech Republic	PCR^9^; MG344771	−
*B. divergens*	Bovid	County Wicklow, Ireland	PCR^7,18^; U16730	−
	Red deer	Libavá, Czech Republic	PCR^9^; MG344838	−
	Red deer	Libavá, Czech Republic	PCR^9^; MG344745	−
	Red deer	Hradiště, Czech Republic	PCR^9^; MG344780	−
	Red deer	Libavá, Czech Republic	PCR^9^	−
	Sika deer	Hradiště, Czech Republic	PCR^9^; MG344769	−
*B. duncani*		American Type Culture Collection (ATCC)		−
*B. gibsoni*	Dog	Dog imported to the Czech Republic from Sri Lanka	PCR^9^	−
*B. microti*	Tick (cat host)	Quebec, Canada	PCR^15^	−
	Tick (dog host)	Manitoba, Canada	PCR^15^	−
	Tick (human host)	Manitoba, Canada	PCR^15^	−
	Tick (human host)	Manitoba, Canada	PCR^15^	−
*B. odocoilei*	Elk	Saskatchewan, Canada	PCR^10,16^; KC460321	+
	Tick (dog host)	Manitoba, Canada	PCR/sequence; MW583459	+
	Tick (dog host)	Nova Scotia, Canada	PCR/sequence; MW583460	+
	Tick (dog host)	New Brunswick, Canada	PCR/sequence; MW583462	+
	Tick (human host)	New Brunswick, Canada	PCR/sequence; MW583458	+
	Tick (human host)	Ontario, Canada	PCR/sequence; MW583461	+
	White-tailed deer	East Texas, USA	PCR^5,7^; U16369	+
*B. odocoilei*	Tick (human host)	Canada	PCR/sequence; MW838239	+
*B. microti* coinfection			PCR^15^	
*B. vulpes* sp. nov.^*^	Brown bear	North Slovakia	PCR^9^	−
	Raccoon	Doupov, Czech Republic	PCR^9^	−
	Raccoon	Doupov, Czech Republic	PCR^9^	−
	Raccoon dog	Mnichovo Hradiště, Czech Republic	PCR^9^	−
	Raccoon dog	Doupov, Czech Republic	PCR^9^	−
*Babesia* sp. ‘deer clade’	Red deer	Libavá, Czech Republic	PCR^9^; MG344793	−
	Red deer	Libavá, Czech Republic	PCR^9^; MG344890	−
	Red deer	Libavá, Czech Republic	PCR^9^; MG344892	−
	Red deer	Libavá, Czech Republic	PCR^9^; MG344898	−
	Sika deer	Hradiště, Czech Republic	PCR^9^; MG344758	−
*Babesia* sp. EU1	Roe deer	Hradiště, Czech Republic	PCR^9^; MG344749	−
	Roe deer	Hradiště, Czech Republic	PCR^9^; MG344756	−
*Theileria cervi*	White-tailed deer	USA	PCR^4^; U97054	−
*T. equi*		USDA reference strain		−

Superscript numbers refer to corresponding references.

* Previously *Theileria annae*.

Each of the 48 samples, used to assess specificity, was analyzed in duplicate using the 306-bp PCR assay. Pellets were resuspended in 30 µL of Tris-EDTA buffer. Prior to each PCR, adequate DNA concentration (50–1,000 ng/µL) was confirmed (NanoDrop spectrophotometer; Thermo Fisher). The primers used for this PCR (Millipore Sigma), with nucleotide sequence listed from 5′ to 3′, were Bab CF (AGGCAGCAACGGGTAACG) and Bab 306R (AATACGGTGACGCAGAAA). As described previously, the primers were designed (Geneious prime; Biomatters) based on the *B. odocoilei* DNA sequence (GenBank U16369), with the forward primer (Bab CF) starting at position 318, and the reverse primer (Bab 306R) ending at position 623.^10^ The annealing site of the reverse primer was in the region of the DNA sequence where the 32-position difference between *B. odocoilei* and *B. divergens* was located.^7,10^ The PCR assay was optimized using a final concentration of 4 mM MgCl_2_ and 1.25 units of Taq polymerase at an annealing temperature of 58.8°C. Amplification was performed in a 50-µL PCR reaction containing 33.2 µL of water, 5 µL of 10 × PCR buffer, 4 µL of 50 mM MgCl_2_, 2.5 µL of Bab CF (20 µM), 2.5 µL of Bab 306R (20 µM), 0.5 µL of 25 µM dNTP, 0.25 µL of Accu-start Taq (5 U/µL; Quantabio), and 2 µL of DNA. A Mastercycler pro (Eppendorf) was used, and the thermocycler parameters were as follows: initial denaturation for 3 min at 96°C, then 40 cycles at 94°C for 10 s, 58.8°C for 30 s, and 72°C for 30 s. The final extension was at 72°C for 10 min, and hold at 10°C.

Seven *B. odocoilei* samples consistently produced a single band of the expected size (306 bp). Only nonspecific binding occurred when the PCR assay was challenged with 40 samples comprising 12 different *Babesia* spp. and 2 *Theileria* spp., with no dominant bands present at the 306-bp mark. One sample originated from a tick coinfected with *B. odocoilei* and *B. microti*. The sample showed the expected dominant band of 306 bp, which was confirmed as *B. odocoilei* by sequencing, and a second pale band of larger size. Interestingly, this finding indicates that the primers preferentially bind to the specific binding sites on the *B. odocoilei* template DNA in samples collected from ticks or animals coinfected with *B. microti*. The second band likely reflected nonspecific binding, as expected with *B. microti* (Fig. 1). With the exception of *B. odocoilei*, there was no consistency in the banding pattern for *Babesia* spp. that had >1 sample available (8 species, 34 samples), reinforcing the nonspecific nature of the primer binding in these cases.

**Figure 1.**
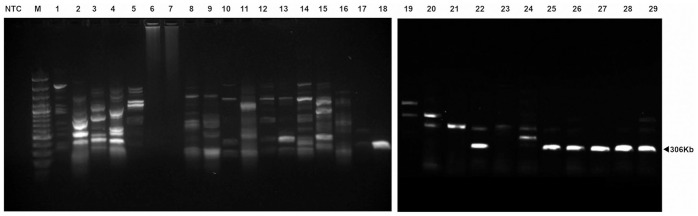
Gel electrophoresis of our PCR assay products using total DNA extracted from samples infected with different species of *Babesia*. In the case of *B. odocoilei*, only a single dominant band (306 bp) was amplified consistently. A secondary larger faint band is observed in samples coinfected with *B. odocoilei* and *B. microti*. In some cases, DNA from samples infected with *B. microti* produce a single band of ~650 bp, but this is not consistent from sample to sample. PCR of template DNA from other *Babesia* spp. resulted in multiple dominant bands of equal intensity and variable size, as well as other multiple faint bands. Lanes: M = marker; NTC = no template control; 1 = *B. canis*; 2–5, 15 = *Babesia* sp. “deer clade”; 6–9, 16 = *B. vulpes*; 10–12 = *B. capreoli*; 13, 14 = *Babesia* sp. EU1; 17 = *B. caballi*; 18, 25–29 = *B. odocoilei*; 19 = *B. duncani*; 20, 21, 23, 24 = *B. microti*; 22 = coinfection with *B. odocoilei* and *B. microti*.

To assess the limit of detection for the PCR assay, DNA was extracted from EDTA-blood from an elk infected with *B. odocoilei* (GenBank KC460321) by digestion using a lysis buffer containing 100 mM NaCl, 500 mM Tris, and 10% sodium dodecyl sulfate. Proteinase K (50 mg) was added, the samples were incubated overnight at 56°C, protein was extracted with phenol–chloroform, and DNA was precipitated with ethanol.^11^ Pellets were resuspended in 30 µL of Tris-EDTA buffer. The DNA concentration of the resulting sample was measured (104 ng/µL; NanoDrop spectrophotometer, Thermo Fisher), and then serially diluted with Tris-EDTA buffer. A 2-µL volume was used, with the total amount of DNA for each dilution as follows: 20.8 ng, 4.16 ng, 0.83 ng, 0.17 ng, 32 pg, 6.4 pg, and 1.28 pg. PCR was performed in duplicate on the original sample, and each dilution, producing a single band of the expected size (306 bp) for all dilutions up to and including the sample with 6.4 pg of DNA. No band was observed for the sample with 1.28 pg DNA (Fig. 2).

**Figure 2.**
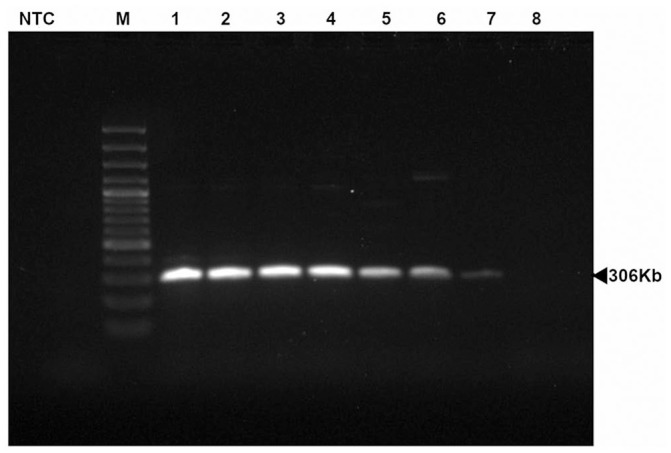
Products of our *Babesia odocoilei* PCR assay performed using 5-fold diluted total DNA extracted from blood samples for the detection of *B. odocoilei*. Lanes: M = marker; NTC = no template control; 1 = 104 ng/µL; 2 = 20.8 ng/µL; 3 = 4.16 ng/µL; 4 = 0.83 ng/µL; 5 = 0.17 ng/µL; 6 = 32 pg/µL; 7 = 6.4 pg/µL; 8 = 1.28 pg/µL.

The PCR assay that we used has been shown to successfully amplify *B. odocoilei* DNA in fresh-frozen tissue, as well as tissue stored in 10% neutral-buffered formalin for up to 6 mo.^10^ This provides an advantage for cases where samples are limited to formalin-fixed tissues, as well as those cases in which tissue processing may be delayed, such as field studies or cases awaiting chronic wasting disease test results. In making the product size of this assay small enough to accommodate formalin-associated protein modifications, the ability to definitively confirm the *Babesia* spp. through sequencing was lost, making the specificity of the primers integral to the utility of this assay. When challenged previously with *B. microti* DNA, only nonspecific binding occurred, and there was no band present at the 306-bp mark.^10^ However, given that cervids can be infected by a number of *Babesia* spp. (including *B. capreoli*, *Babesia* sp. EU1, *B. divergens*, and “deer-clade”),^9^ further investigation into the specificity of this primer set was necessary.

We evaluated here the ability of this PCR to differentiate *B. odocoilei* from 12 other *Babesia* spp. (6 of which infect cervids^8,9^) and 2 *Theileria* spp. (one of which infects cervids^4^). As mentioned, cervid babesiosis is a rare occurrence in Canada, and other infectious disease agents that cause similar clinical signs are not recognized in this geographic region. The species of *Babesia* and *Theileria* used to challenge the specificity of this PCR assay were chosen to reflect those that may be present in cervids alongside *B. odocoilei* in other geographic regions and to exclude cross-reactivity with other similar species regardless of their host specificity or geographic origin. Given that only *B. odocoilei* produced a positive result on the PCR assay, we were able to demonstrate the exclusivity of this assay. The *B. odocoilei* samples that we used originated from 3 different hosts (elk, tick, and white-tailed deer) from a variety of different geographic regions (USA and 6 provinces in Canada), with consistent positive results demonstrating inclusivity within this species of *Babesia*.

Based on our results, it is possible for additional faint, nonspecific bands to accompany the 306-bp dominant band in cases of coinfection, and in these cases further investigation into infection with additional *Babesia* spp. is warranted. Sensitivity was determined to be 6.4 pg of total DNA. Given that our PCR assay is not quantitative, we were unable to determine sensitivity specifically for *Babesia* DNA. Many publications have indicated sensitivity in terms of percentage parasitism. On evaluation of the EDTA-blood smears used in the determination of sensitivity, parasitism was 1.5% of erythrocytes.^16^ The estimated percentage parasitism for the final dilution would be 0.000096%, indicating a highly sensitive PCR. This level of sensitivity is equal to that found for a nested PCR designed to amplify the small subunit rRNA gene for *B. gibsoni.*^2^

Although PCR provides numerous advantages in the detection of infectious disease agents, sample storage conditions can limit performance. The primers that we evaluated have expanded the repertoire of sample types available for *B. odocoilei* testing, and the high sensitivity and specificity of the primers have made this a useful test without the need for confirmation by follow-up sequencing.
